# Genome-wide identification and characterization of reference genes with different transcript abundances for *Streptomyces coelicolor*

**DOI:** 10.1038/srep15840

**Published:** 2015-11-03

**Authors:** Shanshan Li, Weishan Wang, Xiao Li, Keqiang Fan, Keqian Yang

**Affiliations:** 1 State Key Laboratory of Microbial Resources, Institute of Microbiology, Chinese Academy of Sciences, Beijing 100101, People’s Republic of China

## Abstract

The lack of reliable reference genes (RGs) in the genus *Streptomyces* hampers effort to obtain the precise data of transcript levels. To address this issue, we aimed to identify reliable RGs in the model organism *Streptomyces coelicolor*. A pool of potential RGs containing 1,471 genes was first identified by determining the intersection of genes with stable transcript levels from four time-series transcriptome microarray datasets of *S. coelicolor* M145 cultivated in different conditions. Then, following a strict rational selection scheme including homology analysis, disturbance analysis, function analysis and transcript abundance analysis, 13 candidates were selected from the 1,471 genes. Based on real-time quantitative reverse transcription PCR assays, SCO0710, SCO6185, SCO1544, SCO3183 and SCO4758 were identified as the top five genes with the most stable transcript levels among the 13 candidates. Further analyses showed these five genes also maintained stable transcript levels in different *S. coelicolor* strains, as well as in *Streptomyces avermitilis* MA-4680 and *Streptomyces clavuligerus* NRRL 3585, suggesting they could fulfill the requirements of accurate data normalization in streptomycetes. Moreover, the systematic strategy employed in this work could be used for reference in other microorganism to select reliable RGs.

Streptomycetes are famous for their complex developmental life cycles and well-known capabilities to produce secondary metabolites. More than half of naturally occurring antibiotics are produced by this genus^1^. Because of the complex morphogenesis and industrial and medical importance of streptomycetes, the model organism *Streptomyces coelicolor* A3(2) becomes an important subject for basic research, in which investigation of the transcript levels of the target genes is one of a critical step. There are several techniques to analyze transcript levels, such as real-time quantitative reverse transcription PCR (qRT-PCR), microarray, northern hybridization, etc. All these techniques require a reference gene as an internal control to normalize the expression levels of the genes of interest, which avoids potential artifacts caused by sample preparation and detection, and thus providing accurate comparisons of gene expression levels among different samples. Hence, reliable reference genes (RGs) are the prerequisite for accurate measurement of gene expression.

The transcript levels of ideal RGs should keep constant, which are independent of internal and external variations such as life cycle, culture conditions and so on. In addition, their transcript abundances should be similar with those of the target genes^2^. Currently, gene *hrdB* is used as the RG for *S. coelicolor* A3(2) and its derivatives, as well as other *Streptomyces* species. HrdB is the principle sigma factor and represents the primary housekeeping regulator, which differs from the other sigma factors such as HrdA, SigB and WhiG^3^,^4^. However, recent works indicated that the promoter strength of *hrdB* was significantly influenced by culture medium and mutation in *S. coelicolor* M145^5^. In addition, the transcription of *hrdB* was temporally regulated by sigma factor RbpA in *S. coelicolor*^6^ and ShbA in *Streptomyces griseus*^7^, thus suggesting that *hrdB* is not an ideal RG. The 16S rRNA gene is another widely used RG in bacteria^8^,^9^, but it might be not suitable for *S. coelicolor* because of the following drawbacks: first, there are multiple 16S rRNA genes in the genome of *S. coelicolor* A3(2)^10^ and the measured transcripts of 16S rRNA is the sum of all homologs; second, the transcript abundance of 16S rRNA is usually much higher than that of the target genes^11^, which makes it difficult to subtract the baseline value accurately during data analysis; third, some works have reported that the transcription of 16S rRNA is affected by some biological factors such as stringent response^12^,^13^. Therefore, it is necessary to identify and characterize more reliable RGs for *S. coelicolor* A3(2) and its derivatives.

Previously, RGs were normally selected from a set of constitutively expressed genes obtained by qRT-PCR^14^,^15^. Compared to this technique, transcriptome microarray provides gene expression data at the genome scale and thus offers greater potential to mine credible RGs^16^,^17^. To provide reliable RGs for *S. coelicolor* strains, in this work, we applied statistical analysis to four different time-series microarray datasets of *S. coelicolor* and got the first pool containing genes with stable expression profiles. Then thirteen candidate RGs were obtained from this pool by rational selection, and their transcript levels were evaluated based on experimental validation. The top five genes with the most stable transcript levels showed the similar expression profiles in different *S. coelicolor* strains, indicating they are reliable as RGs for this species. Additionally, these five genes also possessed the constant transcript levels in other *Streptomyces* species, which implies their possibilities as RGs in the genus *Streptomyces*.

## Results

### Identification of the first pool containing genes with stable transcript levels

**Figure 1:**
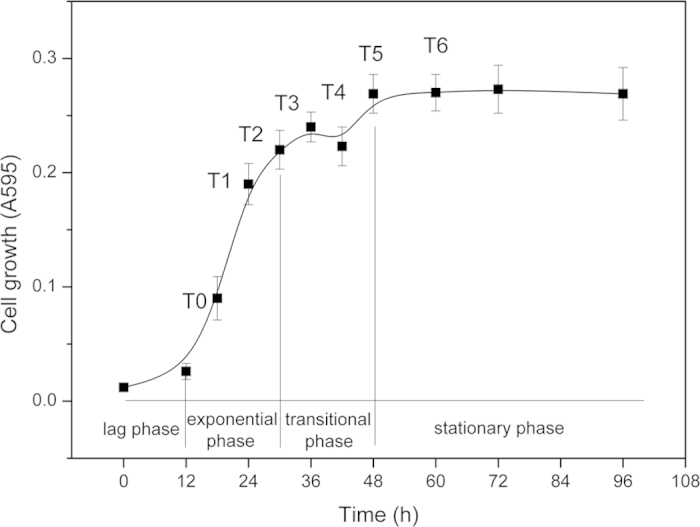
Growth of *S. coelicolor* M145 cultivated in liquid SMM. T0-T6 indicates the time points when cultures were harvested for time-series microarray experiment. Cell growth were determined by diphenylamine colorimetric assay at 595 nm^41^. Data are expressed as average values obtained from three independent experiments.

The ideal RGs should keep the constant transcript levels in different culture conditions. To make sure of that, we accessed the major microarray databases, NCBI Gene Expression Omnibus (GEO) database and Stanford Microarray Database, and extracted three sets of time-series transcriptome microarray data of *S. coelicolor* M145: GSE18489^18^, GSE30569^19^ and GSE2983^20^ (the detailed information are listed in Supplementary Table S1). The experimental conditions of these transcriptome microarrays were quite distinct. The first two datasets were obtained from growth in two different defined fermentation media^18^,^19^, and the last was obtained from growth in the modified R5 rich medium^20^. However, transcriptome microarray describing global gene expression profiles in the minimal medium was not available. To get reliable RGs as possible as we could, we carried out time-series transcriptome microarray experiments of *S. coelicolor* M145 in the liquid supplemented minimal medium (SMM), which is a widely used minimal medium in laboratory. Samples were harvested from seven time points: T0 to T6 corresponding to 18, 24, 30, 36, 42, 48 and 60 h, respectively, covering the exponential, transitional and stationary phase (Fig. 1). The microarray data containing the expression profiles of 7,729 genes were deposited in the GEO database with the accession number GSE53562.

**Figure 2:**
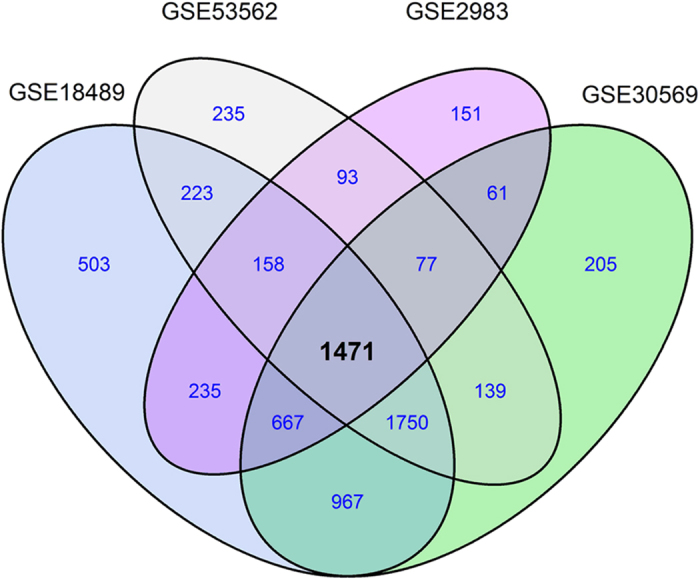
The number of genes with stable transcript levels of *S. coelicolor* cultivated in different media. Venn diagram showing the numbers of genes with the stable expression profiles from four sets of microarray data obtained from growth in different culture media, as well as their intersections.

Global analysis of the four datasets showed there were 6,019, 5,375, 2,990 and 4,145 genes with stable transcript levels in dataset GSE18489, GSE30569, GSE2983 and GSE53562, respectively (Supplementary Dataset S1). The intersection of the four datasets contained 1,471 genes, which could keep the constant expression profiles under different culture conditions (Fig. 2 and Supplementary Dataset S1). These genes were chosen and designated as our first pool of potential RGs.

### Further rational selection of candidate RGs from the first pool

**Figure 3:**
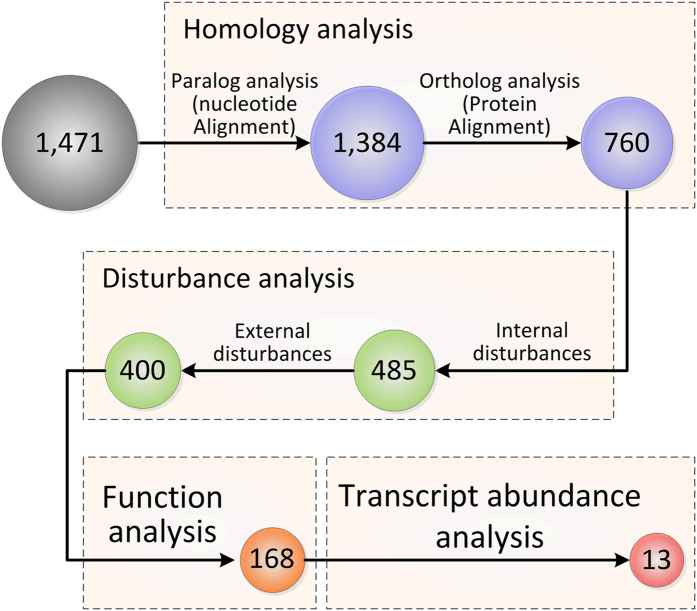
Rational selection workflow of reliable candidate RGs for *S. coelicolor*.

The reliable candidate RGs were further selected from the 1,471 genes by following a strict and rational selection scheme (Fig. 3). The ideal RGs should have no homologous alleles in one genome, or the measured transcript abundance might be the sum of all homologs rather than that of a single gene. Moreover, these RGs had better to be functionally conserved, thus they might be used as RGs in the genus *Streptomyces*. To meet these criteria, the 1,471 stably transcribed genes were first subjected to nucleotide sequence alignment against the genome of *S. coelicolor* A3(2) by BLASTn (see Methods), which excluded 87 genes with more than one paralog in this genome (Supplementary Dataset S2). Next, the corresponding protein sequences of the remaining 1,384 candidate RGs were used as queries to search against the local database containing all the proteins (569,791) of *Streptomyces* species deposited in UniProt by BLASTp (see Methods). This step removed 624 genes without conserved functional roles in streptomycetes (Supplementary Dataset S2). Finally, 760 genes were preserved after homology analysis (Supplementary Dataset S3).

Internal and external disturbance analyses were then performed on the remaining 760 genes to generate candidates with more stable transcript levels. The internal disturbance test was performed by examining the expression profiles of these genes in different mutants of *S. coelicolor* M145, specifically, the Δ*glnK* and Δ*phoP* mutants and their corresponding time-series transcriptome microarray datasets GSE30570 and GSE31068 were used, respectively. The test sequentially excluded 103 and 172 genes, whose expression profiles were strain-specific 
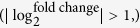
 in the Δ*glnK* and Δ*phoP* mutants, respectively (Supplementary Dataset S2). For external disturbance test, 5 μM of jadomycin B, which can act as an external antibiotic signal to modulate the behaviors of *S. coelicolor*^21^, were used to generate the transcriptome microarray GSE53563. Among the remaining 485 genes, 57 and 28 genes displayed differential transcript levels in jadomycin B treated *S. coelicolor* M145 and its Δ*scbR*2 mutant, respectively, implying they were sensitive to external stress and should be eliminated (Supplementary Dataset S2). There were 400 candidates after disturbance analysis (Supplementary Dataset S3).

The 400 genes were then subjected to predicted function analysis based on Clusters of Orthologous Groups (COGs) assignment, which could help to discard the non-essential genes and keep more reliable candidates with conserved functional roles. Therefore, the genes without COGs classification (78 genes), or falling into the categories of function unknown ([S], 29 genes) and general function prediction only ([R], 49 genes) were all removed first. Then, genes annotated as secondary metabolite biosynthesis/transport/catabolism ([Q], 10 genes) were excluded too, because these genes are usually tightly temporally regulated by physiological or environmental signals and multiple regulators^22^,^23^. Regulators contain diverse protein families, and their encoding genes usually present the growth-phase dependent expression profiles^20^. Thus genes annotated as regulators (66 genes) were also removed here. Taken together, total 232 genes were excluded after the biological function analysis. The remaining 168 qualified genes were listed in Supplementary Dataset S3.

**Figure 4:**
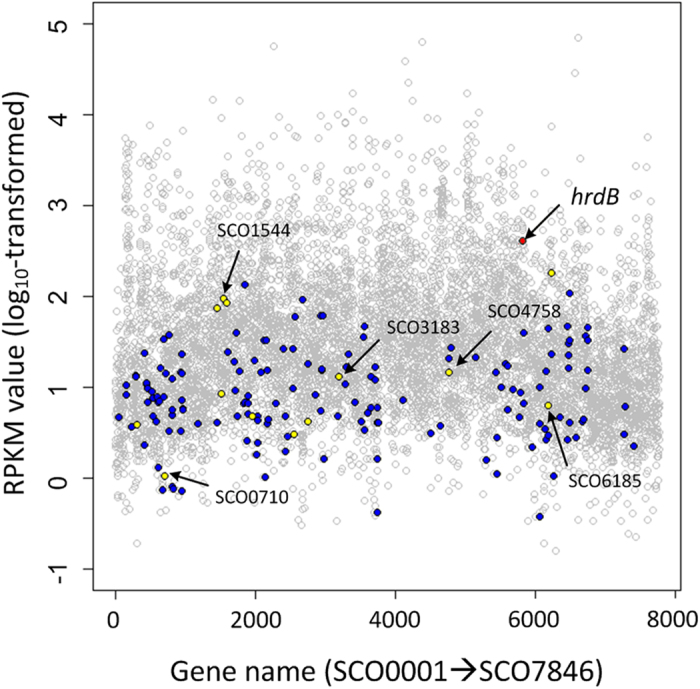
Transcript abundances of genes measured by RNA-Seq in *S. coelicolor* M145. Transcript abundances of all measured genes (gray circles), the 168 qualified candidate RGs (blue dots), the 13 experimentally validated RGs (yellow dots), the five selected RGs (the arrow pointed yellow dots) and the currently used RG hrdB (red dot). Transcript abundance was shown in a log10-transformed scale.

To ensure RGs have comparable transcript levels to the target genes^2^, the transcript abundances described by the values of RPKM (see Methods) of all genes in *S. coelicolor* M145 were analyzed based on RNA-Seq data. As shown in Fig. 4, the transcript levels of the vast majority of genes of *S. coelicolor* M145 were concentrated in the range of 0.5 to 2 (log_10_-transformed RPKM value), few genes showed extremely high or low transcript abundances. This phenomenon might be reasonable, because a robust metabolic network is essential to keep a cell survival, while it is quite difficult to form the balanced pathways or a robust metabolic network with numerous genes showing the dramatically different transcript levels^24^. The range of the transcript abundances of the 168 genes, from −0.42 to 2.25 (log_10_-transformed RPKM value), showed almost 90% coverage of that of all genes in M145 (Fig. 4). To facilitate the experimental validations, we finally selected 13 genes with different functions and comparable transcript levels to most of genes (Table 1 and Fig. 4).

### Evaluating the stabilities of transcript levels of the 13 RGs in *S. coelicolor* M145

**Figure 5:**
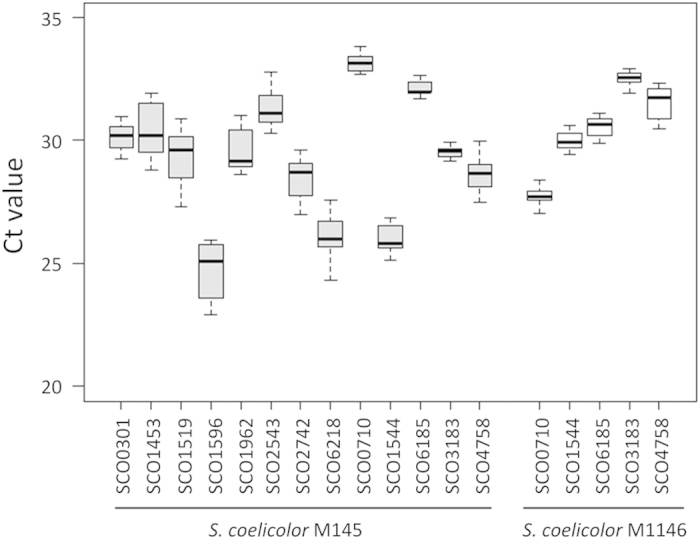
Stabilities of transcript levels of the candidate RGs in different S. coelicolor strains. The Box-and-Whisker plot indicates the range of Ct values from different growth stages of the candidates in *S. coelicolor* M145 and M1146. Two whiskers indicated the maximal and minimal Ct values. The box provides a simple description of a distribution of values by depicting the 25^th^ and 75^th^ percentile values as the bottom and top of a box, respectively. The median is depicted as a line across the box.

The 13 candidate RGs, which possessed stable transcript levels under different culture conditions, were further subjected to experimental confirmation in M145 by real-time qRT-PCR. Samples of M145 were taken from SMM cultures grown at 18, 24, 42 and 60 h. Variation of the transcript levels of each gene was assessed by coefficient of variation (CV, see Methods) according to the threshold cycle (Ct) values of four time points^16^. Here, all CV values of the 13 candidates were less than 0.05 (Supplementary Table S2), suggesting every gene showed the stable transcript levels during the different growth phases we tested. Among them, gene SCO3183 encoding a 5-formyltetrahydrofolate cyclo-ligase showed the lowest CV value of 0.008 and displayed the narrowest dispersion of Ct values (Fig. 5), implying it might have a more constant transcript level than other candidates.

To avoid the potential bias, stabilities of the transcript levels of the 13 genes were further assessed by three different algorithms GeNorm^11^, NormFinder^25^ and BestKeeper^26^ simultaneously. As shown in Table 2, all genes showed the stable transcript levels as their stability values all within the individual threshold of each algorithm (see Methods), except for gene SCO1596 (a putative osmosensitive K^+^ channel histidine kinase) that exceeded the upper limit of stability value recommended by BestKeeper. The stability orders of genes ranked by the three algorithms showed minor differences (Table 2), which mainly ascribed to the distinct analytical principles of these algorithms^14^. The top five genes in the combined results of the three algorithms were SCO0710, SCO6185, SCO1544, SCO3183 and SCO4758, which were almost the same as those suggested by geNorm, NormFinder and BestKeeper (Table 2). Therefore, they were finally selected as the candidate RGs.

According to COGs, SCO0710 is responsible for amino acid transport and metabolism. SCO6185 encodes a glycosyltransferase, which is closed to the biosynthesis of cell wall^27^. SCO4758 encodes a conserved protein, S-adenosyl-L-methionine:tRNA (uracil-5-)-methyltransferase, which might be essential for the viability of streptomycetes^28^. SCO1544 encodes a putative protein disulfide isomerase (PDI), and it is highly conserved in streptomycetes. As PDI is an essential catalyst in protein folding^29^, SCO1544 might play a critical role in this genus. SCO3183 encoding a 5-formyltetrahydrofolate cyclo-ligase is unique in *S. coelicolor* and very conservative in 63 different *Streptomyces* species as analyzed by protein alignment, implying the biological function of SCO3183 might be indispensable. Considering the putative important roles of the five genes, maybe it was reasonable to choose them as RGs for *S. coelicolor*.

### Evaluating the stabilities of transcript levels of the five RGs in *S. coelicolor* M1146

To ensure the five candidate RGs are applicable in *S. coelicolor*, stabilities of their transcript levels should be evaluated in different *S. coelicolor* strains. In this work, *S. coelicolor* M1146 was chosen, which is derived from M145 by removing the biosynthetic clusters of actinorhodin (ACT), prodiginines (RED), calcium-dependent antibiotic (CDA) and the type I yellow *coelicolor* polyketide (yCPK)^30^. Since M1146 showed the obviously different metabolic profiling from M145^30^, this strain could facilitate us to evaluate the stability of transcript levels of the five candidates under complex metabolic and regulatory changes. The five genes presented diverse transcript levels in different strains, whereas all of them displayed the constant profile of transcript levels (Fig. 5 and Supplementary Table S2). These results imply that changes of metabolism in *S. coelicolor* might have minor influence on the stabilities of the transcript levels of the five RGs. Hence, the five genes could be used as reliable RGs in *S. coelicolor*.

### Profiles of transcript levels of the five RGs in other *Streptomyces*

**Figure 6:**
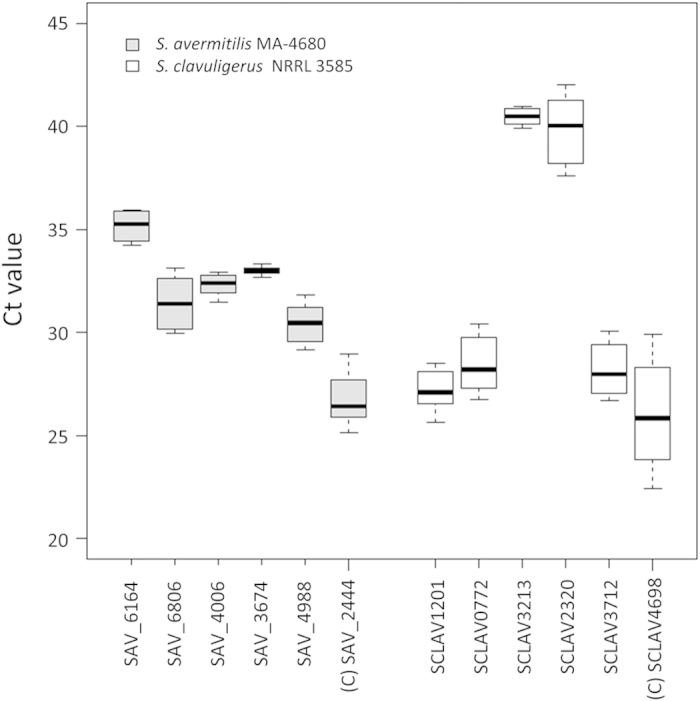
Profiles of transcript levels of the five RGs in other *Streptomyces*. The Box-and-Whisker plot indicates the range of Ct values from different growth stages of the orthologs of the five selected RGs of *S. coelicolor* in *S. avermitilis* MA-4680 and *S. clavuligerus* NRRL 3585, respectively. Details of the Box-and-Whisker plot were as described in Fig. 5. Genes listed from left to right were the corresponding orthologs of genes in M1146 (Fig. 5) with the same order. The capital letter c in the bracket indicates the orthologs of *hrdB*.

The five selected RGs are highly conserved in *Streptomyces* species, as least in those with complete genomes (Supplementary Table S3). To test whether the five genes could keep the constant transcript levels in addition to *S. coelicolor*, their corresponding orthologs were subjected to real-time qRT-PCR assays in *Streptomyces avermitilis* MA-4680 and *Streptomyces clavuligerus* NRRL 3585, which are the producers of two kinds of important antibiotics avermectins and clavulanic acid, respectively. Four samples of each strain were taken from early- and mid-exponential, transitional and stationary phase based on their respective cell growth curves (Supplementary Figure S1). Each of the five RGs showed different transcript abundances under different genetic backgrounds (Figs 5 and 6), whereas these RGs could keep the constant transcript levels in various conditions (CV<0.05) (Supplementary Table S2). In *S. clavuligerus* NRRL 3585, dispersions of the Ct values depicted by the Box-and-Whisker plot of the five selected RGs were all more concentrated than the control gene SCLAV4698 (the ortholog of *hrdB*) (Fig. 6), which were also in consistent with their individual variation of Ct values described by CV (Supplementary Table S2). Similar results were also found in *S. avermitilis* MA-4680. Although gene SAV_6806 (the ortholog of SCO1544) showed the almost equal dispersion of Ct values as that of the control gene SAV_2444 (the ortholog of *hrdB*) (Fig. 6), its CV value was less than that of the control (Supplementary Table S2), implying SAV_6806 had a more constant level of transcript abundances.

## Discussion

RGs are commonly selected from housekeeping genes, which is a category of genes with essential cellular functions and presumptive invariable expression^31^, such as 16S rRNA and *rpoB* (the RNA polymerase β subunit gene) genes in *Escherichia coli*^32^ and other bacteria. For streptomycetes, the essential sigma factor gene *hrdB* is transcribed in all growth stages and this gene is conventionally used as the RG. However, the transcription of *hrdB* has been proved to be regulated by several factors and its transcript levels were not always constant^6^,^7^, implying the essential genes might not be the best RGs in the genus with complex life cycles and metabolism changes. One of a possible reasons might be that the complex regulatory networks dynamically control the essential genes at different growth stages^1^. Hence, it might be more feasible to get the ideal RGs for the genus with complex life cycles by screening them from a group of genes with the constant expression profile under various growth stages and culture conditions. Based on this consideration, we implemented meta-analysis of various transcriptome data and experimental validation to obtain five reliable RGs for *S. coelicolor*. This systematical and rational approach could be used for reference to identify RGs in other organisms.

Numerous transcriptome data of *S. coelicolor* deposited in the public databases offer us an opportunity to mine potential RGs^8^,^16^,^17^. We extracted all the time-series transcriptome microarray datasets of *S. coelicolor* M145 obtained in different conditions, and supplied an extra set of data in minimal media that was not available in the public databases. Therefore, it is possible for us to investigate the expression profiles of genes in different types of culture conditions: the minimal media, the rich media and the fermentation media. The significant discrepancies among these conditions were conducive to screen the genes with highly constant transcript levels in various conditions, which is a prerequisite to obtain reliable RGs.

When selecting RGs from a pool of candidates with the constant transcript levels, CV is always the primary criterion^16^,^33^, which is the standard to measure the stability of gene expression profile. However, depending on this single standard might bring some potential risk, because some genes might fall into some functional categories in which the genes show significantly differential expression profiles in general. For instance, *S. coelicolor* M145 contains more than 20 secondary metabolite clusters^10^, while it was hard for genes involved in these clusters to keep the stable transcript levels in different conditions (Supplementary Figure S2)^18^. One of a primary reason might be the physiological or environmental signals and multiple regulators that tightly regulated the transcription of genes related to secondary metabolism^21^,^22^,^23^,^34^. Another arresting group was the category of regulatory genes. The genome of *S. coelicolor* contains as many as 965 genes annotated as a regulator^10^,^35^, while less than 10% of them (74 genes) maintained the stable transcript levels in the tested conditions (Supplementary Dataset S3). Previous researches had demonstrated the transcription of regulatory genes is usually condition-dependent^20^ and regulated by a highly dynamic complex regulatory network of *S. coelicolor*, which makes it difficult to ensure whether the 74 genes remain stable in other conditions^35^. Thus, the functions of genes were carefully considered in addition to CV in our selection workflow, which helped to eliminate the trustless genes and increase the reliability of the selected RGs.

Gene *hrdB* has been used as RG for decades and the wrong result arisen from it has not been found so far. The transcript levels of *hrdB* were not always constant, and a shift primarily occurred in the transitional phase (approximately during 36 to 40 h, Supplementary Figure S3). However, *hrdB* could keep the constant transcript levels in exponential phase and stationary phase, respectively. These indicate that the transcription of *hrdB* was culture condition- and growth-dependent^5^, ^6^, ^7^. Thus in some particular cases, using *hrdB* as the internal control might not affect the final results.

Currently, the use of multiple RGs is deemed as the preferred method to obtain precise gene expression data^11^. The transcript abundances of these five new reliable RGs selected in this work were different, and they were comparable with the transcript abundances of about 90% of the total genes in the genome of *S. coelicolor*. Therefore, these five genes could fulfill the requirement of more accurate data normalization by multiple RGs.

## Methods

### Strains and growth conditions

*S. coelicolor* M145^10^ and its derivative M1146^30^ were kindly provided by Professor Mervyn Bibb of John Innes Centre. *S. avermitilis* MA-4680^36^ and *S. clavuligerus* NRRL 3585^37^ were from our laboratory collection. To prepare spore suspensions of *S. coelicolor* strains and *S. avermitilis* MA-4680, mannitol-soya flour (MS) agar was used to grow these strains^38^, while solid yeast dextrose (YD) agar was used to make spore suspension of *S. clavuligerus* NRRL 3585^39^. All agar plates were cultivated at 28 °C. For fermentations of *S. coelicolor* strains, the fresh spores were inoculated into 250 Erlenmeyer flasks containing 100 ml liquid SMM and cultivated at 28 °C, 250 rpm for 96 h^38^. Fermentations of *S. avermitilis* MA-4680 were implemented by inoculating spores in liquid yeast extract-malt extract (YEME) medium^40^ and cultivated at 28 °C, 250 rpm for 144 h. Prior to the fermentations of *S. clavuligerus* NRRL 3585, the spores were inoculated in 50 ml liquid YD medium in 250 ml Erlenmeyer flasks and shaken at 28 °C, 250 rpm for 48 h to prepare the seed cultures of this strain. For fermentations, the seed cultures were inoculated into the soybean medium^41^ with a dilution ratio of 1:20, and cultivated for 108 h at the same condition as that of the seed preparation. For all strains, the final concentration of spore inoculation was 4 × 10^6^ spores/ml^38^.

### Measurement of cell growth

Cell growth was monitored by using the diphenylamine colorimetric method^41^. For growth quantification, cell pellets were harvested from 1 ml cultures by centrifugation at 10,000 × *g* at room temperature for 10 min and washed twice with the appropriate buffer. The cell pellets were then resuspended with 2 ml diphenylamine reagent and incubated at 60 °C for 1 h. After centrifugation, the supernatants were transferred to 96-well microtiter plate and measured at 59  nm by a multifunctional microtiter plate reader (Synergy Hybrid Reader, BioTek, USA).

### RNA extraction and quantification

Cell pellets were quickly harvested by fast filtration and flash frozen in liquid nitrogen, and then ground into powder. RNA was isolated by using Ambion RiboPure bacteria kit (Life Technologies, USA) according to the manufacturer’s instructions. Total RNA was purified by NucleoSpin® RNA clean-up kit (MACHEREY-NAGEL, Germany). The integrity and quantity of isolated RNA were checked by denaturing agarose gel eletrophoresis and NanoDropND-1000 spectrophotometer (NanoDrop Technologies, USA), respectively.

### Microarray experiments

To obtain the time-series gene expression profiles of *S. coelicolor* M145 in the minimal medium, the SMM cultures were sampled from 18, 24, 30, 36, 42, 48 and 60 h. To investigate the effect of external disturbance on gene expression profiles, 5 μM jadomycin B that could act as an extracellular signal^21^ was supplemented in SMM medium at the beginning of fermentation. Then the cultures were sampled at 30 h for transcriptome microarray experiments. The transcription array (12 × 135 k) was manufactured based on NimbleGen proprietary Maskless Array Synthesizer (MAS) technology. For each of them, DNA microarrays include 7,729 target genes from *S. coelicolor* M145 fixed on glass slides; each gene is represented by up to six unique probes consisting of 21-mer synthetic oligonucleotides designed by OligoArray 2.1 software based on the genome of *S. coelicolor* A3 (2) [GenBank: NC_003888]. Reverse transcription and amplification of total RNA were performed using Ambion MessageAmp II- Bacteria kit (Life Technology, USA) as described by the manufacturer’s instructions. Then, 5 μg of amplified RNA was reverse transcribed with random primer, and the cDNA was labeled with fluorescent dyes (Cy3) using Klenow enzyme. After suspending the labeled samples, the transcription arrays were hybridized with the hybridization kit (Roche NimbleGen). The arrays were scanned using MS200 scanner (NimbleGen) with 2 μm resolution. The probe intensities were subjected to background correction, quantile normalization^42^, and gene expression data were generated using Robust Multi-Array Analysis algorithm^43^,^44^ with the Nimble Scan Software, version 2.6 (Roche NimbleGen, Inc.).

### RNA sequencing (RNA-Seq)

The SMM cultures of *S. coelicolor* M145 sampled at 24 h were used for massively parallel cDNA sequencing to present a genome-wide map of transcript levels. Details of the RNA-Seq method are previously described^45^. The cDNA libraries were prepared and analyzed on Illumina HiSeq 2000 platform. To obtain appropriate deep sequencing results, samples were sequenced at least twice. The 120-bp raw PE reads were first processed by the FASTX-Toolkit (http://hannonlab.cshl.edu/fastx_toolkit/) to remove the low quality (phred quality < 5) reads with sequencing adaptors. Then, the Burrows-Wheeler Aligner’s Smith-Waterman Alignment (BWA-SW) program was used to align the remaining reads to the edited genome sequence of *S. coelicolor*. Picard tools (http://picard.sourceforge.net/) were used to map the total number of reads to each gene. The transcript abundance of each gene in prokaryotes could be denoted by the value of RPKM as described in the following formula^46^,^47^.


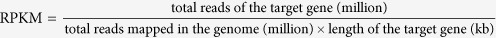


### Real-time qRT-PCR

*S. coelicolor* M145 and M1146 sampled at 18, 24, 42 and 60 h were used for real-time qRT-PCR experiments. All primer pairs (Supplementary Table S4) were designed based on genome sequence of *S. coelicolor* [GenBank: NC_003888]. Amplification efficiencies and correlation coefficients (*R*^*2*^) were generated using the slopes of the standard curves obtained from serial dilutions. Standard curves with an appropriate fold dilution series were used to calculate the amplification efficiency with the following formula: efficiency (%) = (10^(−1/slope)^−1) × 100. The *R*^*2*^ values of all standard curves reached 0.99, and the amplification efficiencies were all above 90%. For experiments, the first-stand cDNA was synthesized using quantitative DNaseI treated RNA template (1 μg) and random primers. Subsequently, each 20 μl reaction system contained 10 μl UltraSYBR Mixture (2×) (With Rox, Cwbio. Co. Ltd, China), 0.4 μl of each primer (10 μM) and 2 μl 10-fold diluted cDNA template. The reaction parameters were as follows: 95 °C for 10 min, followed by 45 three-step amplification cycles consisting of denaturation at 95 °C for 15 s, annealing at 55 °C for 30 s and extension at 72 °C for 30 s. The amplification specificity of each assay was confirmed by melting curve analysis carried out at 60–95°C. After running on an ABI7500 Real-Time PCR System (Applied Biosystems, USA), results were collected and analyzed using the supporting 7500 software (v2.0.4). Experiments were performed using three biological replicates.

### Bioinformatics analysis

Nucleotide and protein alignments were generated using a local BLAST+ v 2.2.28 installation. The genome of *S. coelicolor* A3(2) and all protein sequences of *Streptomyces* species were obtained from NCBI (http://www.ncbi.nlm.nih.gov/, Jun 2013) and UniProt (http://www.uniprot.org, Jun 2013), respectively. Paralogs of a target gene were searched by aligning the nucleotide sequence of the target gene against the genome of *S. coelicolor* A3(2) with BLASTn. Orthologs of the interested protein were searched by aligning the amino acid sequence of this protein against the local database containing all proteins of *Streptomyces* species from UniProt with BLASTp. If the orthologs of a target protein could be found in more than twenty different *Streptomyces* species, this protein was supposed to have a conserved biological function in this genus. The parameters for both nucleotide and protein alignment were set with an E-value of 1e–5, query coverage of 0.7 and identity of 0.7^48^. For biological function analysis, genes of *S. coelicolor* were classified according to their functions annotated by COGs database^49^.

### Statistical analysis

Analyses of gene expression profiles and gene transcript abundances were implemented by R statistical software (Ri386 3.0.1). For each time-series transcriptome microarray dataset, the expression signals of each gene from different time points were normalized to that obtained from the first sampling time point, generating a series of fold changes of gene expression. If the all fold changes (increased or decreased) of a gene were no more than two 
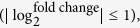
 the transcript level of this gene was thought to be constant. CV 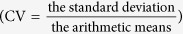 and the Box-and-Whisker plot^16^ were used to describe the variation and dispersion of the transcript levels of a gene based on the Ct values generated from real-time qRT-PCR. Generally, the value of CV less than 0.1 indicates the difference among a set of data is quite small. The lower of CV values, the more stable of the transcript levels. Stabilities of the transcript levels of the candidate RGs were evaluated by three different algorithms, geNorm^11^, NormFinder^25^ and BestKeeper^26^. The geNorm, NormFinder and BestKeeper recommend the upper limit of stability values for a gene with a stable transcript level are 1.5^11^, 1.5^25^ and 1.0^26^, respectively.

### Microarray data accession number

The microarray data obtained in this work are available at the GEO with the accession numbers of GSE53562 and GSE53563. The other transcriptome microarrays could also be obtained in the GEO database (http://www.ncbi.nlm.nih.gov/gds).

## Additional Information

**How to cite this article**: Li, S. *et al.* Genome-wide identification and characterization of reference genes with different transcript abundances for *Streptomyces coelicolor. Sci. Rep.* **5**, 15840; doi: 10.1038/srep15840 (2015).

## Supplementary Material

Supplementary Information

Supplementary Dataset 1

Supplementary Dataset 2

Supplementary Dataset 3

## Figures and Tables

**Table 1:** Thirteen candidate RGs in *S. coelicolor* M145.

Gene	COG^a^	Biological function	Transcriptabundance^b^
SCO1453	[C]	Coenzyme F420-dependent N5,N10-methylene tetrahydromethanopterin reductase and related flavin-dependent oxidoreductases	1.87
SCO0710	[E]	ABC-type branched-chain amino acid transport systems, ATPase component	0.02
SCO0301	[G]	Gluconolactonase	0.59
SCO6218	[G]	Fructose-2,6-bisphosphatase	2.25
SCO3183	[H]	5-formyltetrahydrofolate cyclo-ligase	1.12
SCO4758	[J]	SAM-dependent methyltransferases related to tRNA (uracil-5-)-methyltransferase	1.16
SCO2742	[K]	DNA-directed RNA polymerase specialized sigma subunit, sigma 24 homolog	0.62
SCO1519	[L]	Holliday junction resolvasome, DNA-binding subunit	0.93
SCO2543	[M]	Dihydrodipicolinate synthase/N-acetylneuraminate lyase	0.49
SCO6185	[M]	Glycosyltransferase	0.80
SCO1544	[O]	Protein-disulfide isomerase	1.97
SCO1962	[P]	Ca^2+^/H^+^ antiporter	0.68
SCO1596	[T]	Osmosensitive K^+^ channel histidine kinase	1.93

^a^The function of different COG categories are as follows: [C] Energy production and conversion, [E] Amino acid transport and metabolism, [G] Carbohydrate transport and metabolism, [H] Coenzyme transport and metabolism, [J] Translation, ribosomal structure and biogenesis, [K] Transcription, [L] Replication, recombination and repair, [M] Cell wall/membrane/envelope biogenesis, [O] Posttranslational modification, protein turnover, chaperones, [P] Inorganic ion transport and metabolism, [T] Signal transduction mechanisms.

^b^Gene transcript abundance was represented by log_10_-transformed RPKM value.

**Table 2:** Stabilities of transcript levels of the 13 candidate RGS evaluated by three different algorithms.

Ranking	geNorm		NormFinder		BestKeeper		Combinedresult^d^
	Genename	StabilityValue^b^	Genename	StabilityValue^b^	Genename	StabilityValue^c^	
1	SCO6185 | SCO0710^a^	0.381	SCO1544	0.282	SCO3183	0.2	SCO0710
2			SCO0710	0.368	SCO6185	0.26	SCO6185
3	SCO3183	0.431	SCO6185	0.431	SCO0710	0.31	SCO1544
4	SCO1544	0.53	SCO4758	0.464	SCO0301	0.43	SCO3183
5	SCO4758	0.586	SCO3183	0.474	SCO1544	0.5	SCO4758
6	SCO1962	0.666	SCO1962	0.654	SCO0710	0.53	SCO1962
7	SCO1453	0.728	SCO1453	0.431	SCO6185	0.63	SCO1453
8	SCO1596	0.779	SCO1596	0.53	SCO4758	0.64	SCO0301
9	SCO1519	0.803	SCO6218	0.859	SCO2742	0.72	SCO2543
10	SCO2543	0.838	SCO2543	0.885	SCO1962	0.75	SCO1596
11	SCO0301	0.882	SCO1519	0.918	*hrdB*	0.86	SCO6218
12	SCO6218	0.926	SCO0301	0.928	SCO1519	0.92	SCO1519
13	SCO2742	0.97	SCO2742	0.98	SCO1453	0.96	SCO2742
14	*hrdB*	1.001	*hrdB*	0.984	SCO1596	1.01	*hrdB*

^a^geNorm finally generates a pair of genes with the most stable transcript levels^11^, thus SCO6185 and SCO0710 listed in the same line.

^b^Stability values of geNorm and NormFinder were the calculated M values^11^,^25^ by the two algorithms, respectively.

^c^Stability value of BestKeeper is the standard deviation of the Ct values.

^d^The combined result was obtained with an online tool (http://www.leonxie.com/referencegene.php) by using Ct values.
