# Supplementary material for: Genome-wide identification and characterization of reference genes with different transcript abundances for Streptomyces coelicolor
Source: Sci Rep. 2015 Nov 3;5:15840. doi: 10.1038/srep15840 (PMC4630627; doi:10.1038/srep15840)
Supplement: Supplementary Information [file srep15840-s1.pdf]

Supplementary information

Genome-wide identification and characterization of reference genes with different transcript abundances for *Streptomyces coelicolor*

Shanshan Li†, Weishan Wang†, Xiao Li, Keqiang Fan, Keqian Yang*

State Key Laboratory of Microbial Resources, Institute of Microbiology, Chinese Academy of Sciences, Beijing 100101, People's Republic of China

* To whom correspondence should be addressed. Email: yangkq@im.ac.cn.

† These authors contributed equally to this work

Supplementary Figures

Figure S1 Growth curve of *S. avermitilis* MA-4680 and *S. clavuligerus* NRRL 3585.

Figure S2 Time-series expression profiles of genes in the biosynthetic clusters of secondary metabolites in *S. coelicolor* M145 cultivated in liquid SMM (GSE53562).

Figure S3 Time-series expression profiles of *hrdB* in four microarrays obtained from growth in different culture conditions.

Supplementary Tables

Table S1 Overview of all transcriptome microarray datasets used in the present work.

Table S2 Values of coefficient of variance (CV) of the tested candidates in different *Streptomyces* strains.

Table S3 Orthologs of the five selected RGs in other *Streptomyces* species with complete genome.

Table S4 List of primers used for real-time qRT-PCR in the present work.

Supplementary Data Sets

Dataset S1 Genes with stable transcript levels in the four time-series microarray datasets and their intersection.

Dataset S2 The disqualified genes generated in each selection step.

Dataset S3 Candidate RGs obtained in each rational selection step from the first pool of candidates.

References

Supplementary figures

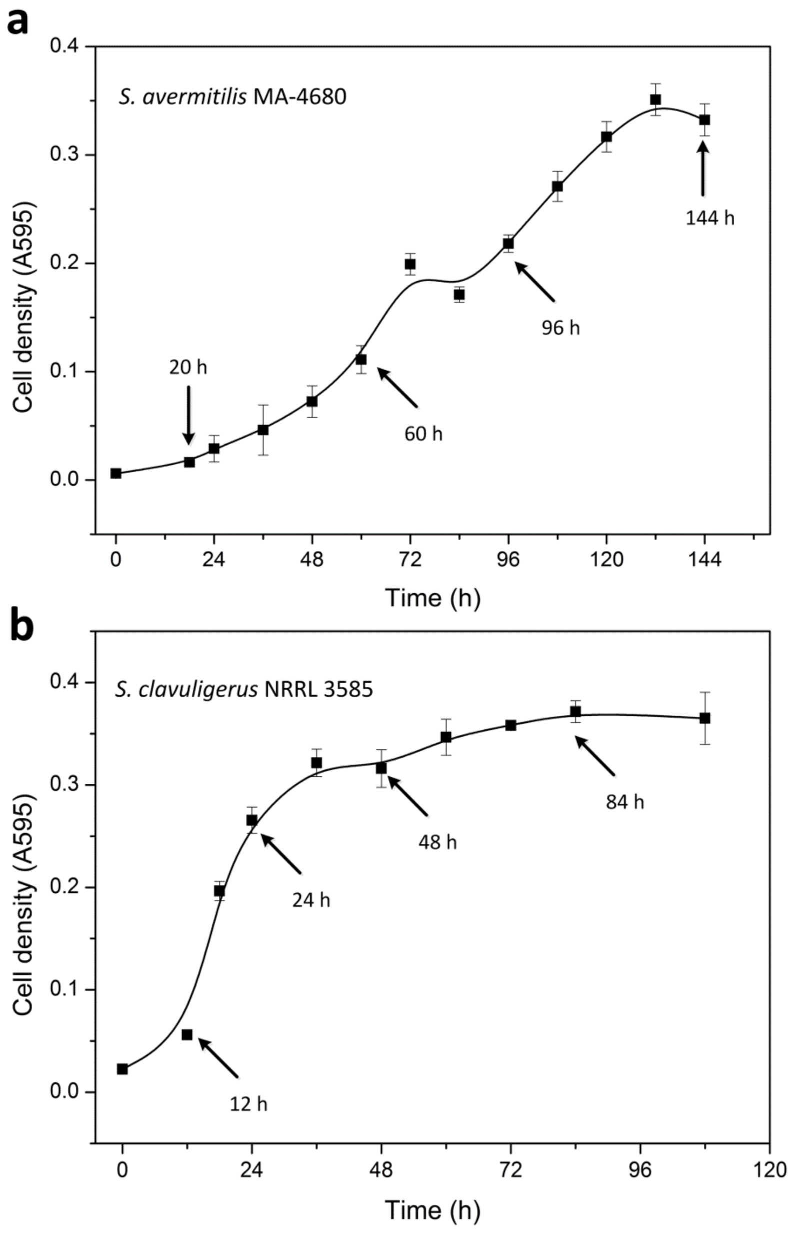

Figure S1 Growth curve of *S. avermitilis* MA-4680 (**a**) and *S. clavuligerus* NRRL 3585 (**b**). Cell growth was determined by diphenylamine colorimetric assay at 595 nm[1]. Arrows indicate the sampling time points for real-time qRT-PCR.

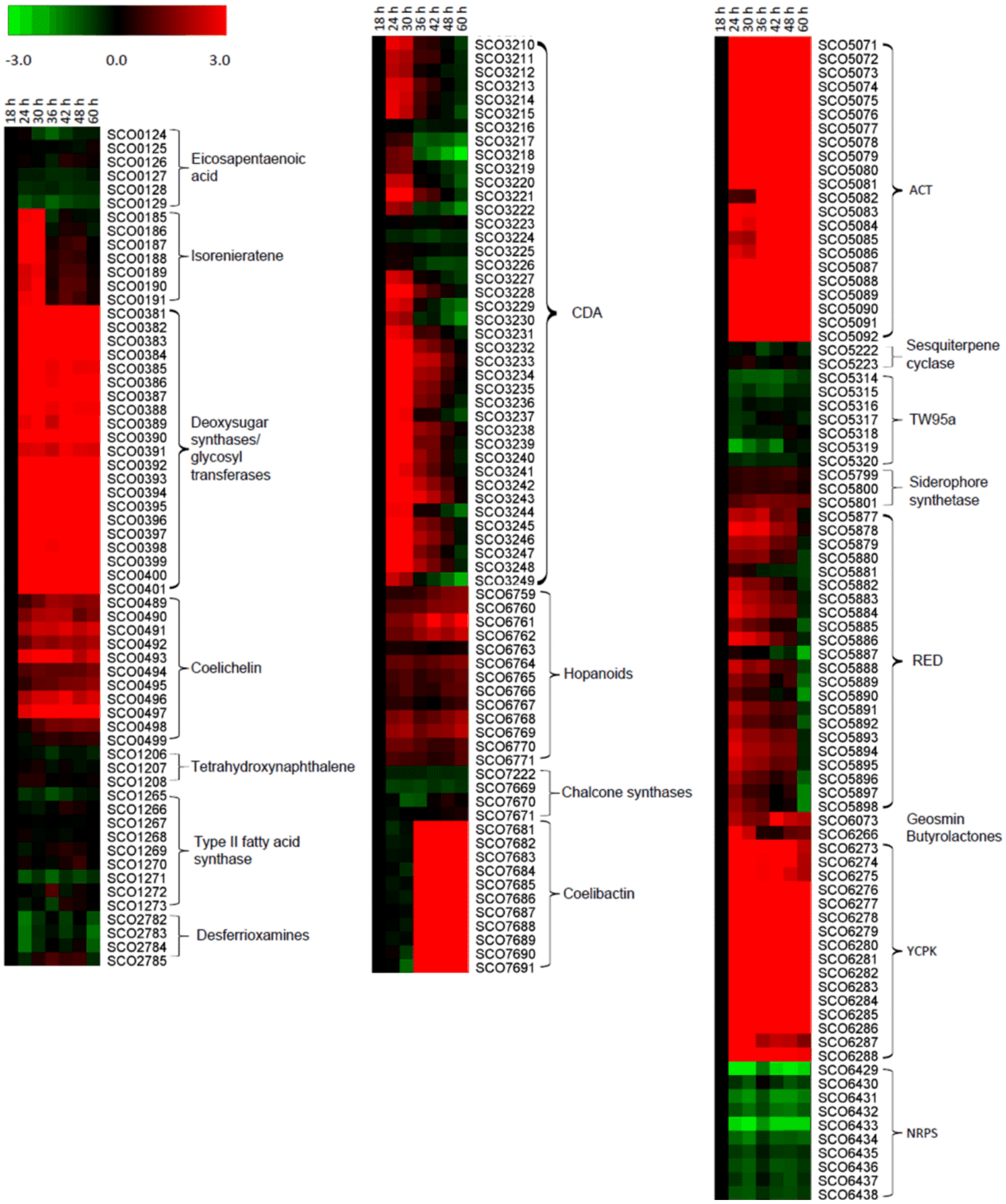

Figure S2 Time-series expression profiles of genes in the biosynthetic clusters of secondary metabolites in *S. coelicolor* M145 cultivated in liquid SMM (GSE53562).

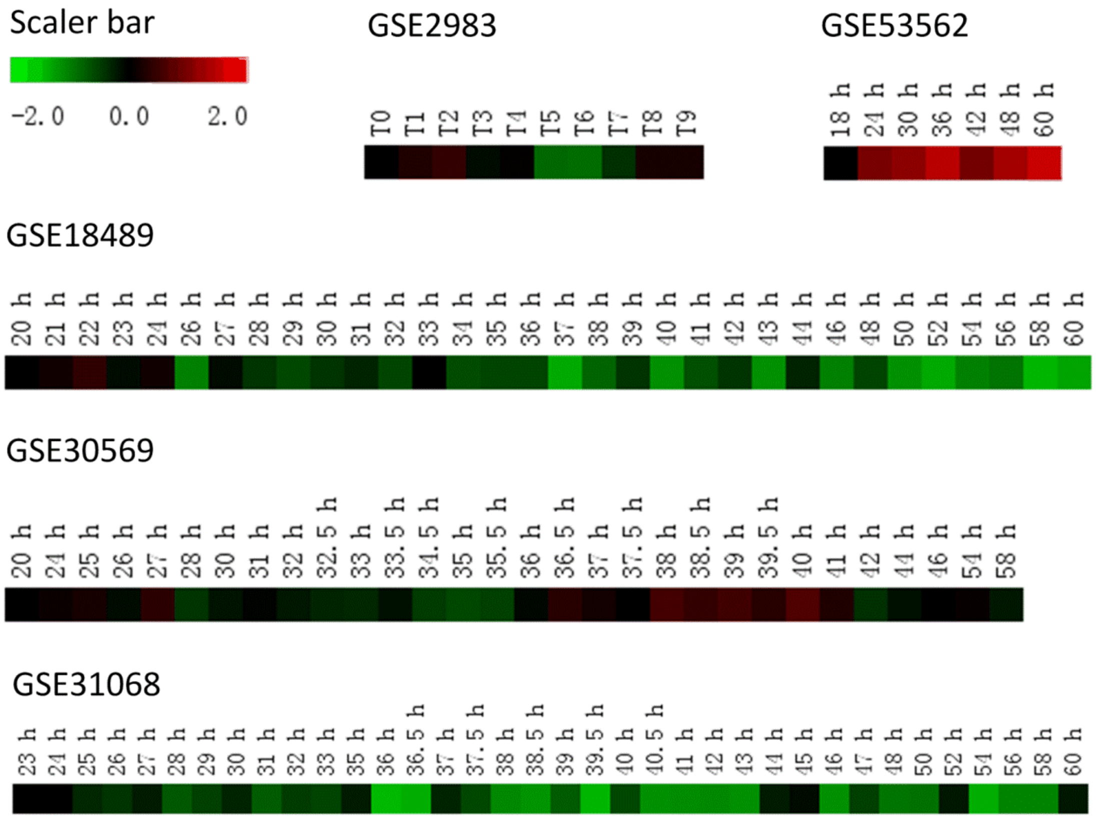

Figure S3 Time-series expression profiles of *hrdB* in four microarrays obtained from growth in different culture conditions. Gene *hrdB* shows unstable expression profiles in dataset GSE18489, GSE31068 and GSE53562. Detailed sampling time of dataset GSE2983 was described by Huang et al.[2].

Supplementary Tables

Table S1 Overview of all transcriptome microarray datasets used in the present work

Microarray	Strain	Culture medium (liquid)	Number of sampling time points	Number of genes[a]	Source
GSE2983	*S. coelicolor* M145	Modified R5 medium	10	4,960	Huang et al.[2]
GSE18489	*S. coelicolor* M145	fermentation medium	32	7,893	Nieselt et al.[3]
GSE30569	*S. coelicolor* M145	SSBM-E	30	7,893	Waldvogel et al.[4]
GSE53562	*S. coelicolor* M145	SMM	7	7,729	This study
GSE30570	The *glnK* mutant (SCglnK-3) of *S. coelicolor* M145	SSBM-E	16	7,893	Waldvogel et al.[4]
GSE31068	The *phoP* mutant (INB201) of *S. coelicolor* M145	The same as GSE18489	36	7,893	Thomas et al.[5]
GSE53563	*S. coelicolor* M145 and its *scbR*2 knock-out mutant	SMM +/- jadomycin B	1	7,729	This study

[a] The number of genes in the chromosome of *S. coelicolor* M145.

Table S2 Values of coefficient of variance (CV) of the tested candidates in different *Streptomyces* strains

S. coelicolor	M145	M1146	*S. avermitilis* MA-4680		*S. clavuligerus* NRRL 3585	
Gene[a]	CV	CV	Gene	CV	Gene	CV
SCO3183	0.008	0.010	SAV_3674	0.006	SCLAV2320	0.042
SCO6185	0.010	0.013	SAV_4006	0.015	SCLAV3213	0.010
SCO0710	0.012	0.013	SAV_6164	0.021	SCLAV1201	0.036
SCO1544	0.022	0.012	SAV_6806	0.041	SCLAV0772	0.048
SCO4758	0.026	0.021	SAV_4988	0.031	SCLAV3712	0.047
SCO0301	0.017					
SCO2543	0.024					
SCO1962	0.030					
SCO2742	0.032					
SCO6218	0.035					
SCO1453	0.036					
SCO1519	0.041					
SCO1596	0.048					
hrdB	0.052	0.029	SAV_2444	0.051	SCLAV4698	0.103

[a] Genes listed in the same row are the orthologs.

Table S3 Orthologs of the five selected RGs in other *Streptomyces* species with complete genome

Strain[a]	Orthologs of the five selected RGs (indicated by locus tag) [b]				
Streptomyces coelicolor A3(2)	SCO0710	SCO1544	SCO6185	SCO3183	SCO4758
Streptomyces avermitilis MA-4680	SAV_6164	SAV_6806	SAV_4006	SAV_3674	SAV_4988
Streptomyces clavuligerus NRRL 3585	SCLAV1201	SCLAV0772	SCLAV3213	SCLAV2320	SCLAV3712
Streptomyces ablus J1074	XNR_4871	XNR_5311	XNR_3380	XNR_2130	XNR_3821
Streptomyces venezuelae ATCC 10712	SVEN_1383	SVEN_1144	SVEN_3952	SVEN_3028	SVEN_4465
Streptomyces sp. PAMC26508	F750_5935	F750_1331	F750_3943	F750_3034	F750_4352
Streptomyces sp. SirexAA-E	SACTE_5642	SACTE_0948	SACTE_6037	SACTE_2644	SACTE_4052
Streptomyces bingchenggensis BCW-1	SBI_07973	SBI_02423	SBI_05009	SBI_06485	SBI_04791
Streptomyces cattleya NRRL 8507	SCAT_RS29700	SCAT_RS33190	SCAT_RS32480	SCAT_RS18420	SCAT_RS25550
Streptomyces collinus Tu 365	B446_RS10440	B446_RS07875	B446_RS04785	B446_RS16505	B446_RS22575
Streptomyces davawensis JCM 4913	BN159_RS04320	BN159_RS34915	BN159_1473	BN159_5085	BN159_RS17765
Streptomyces pratensis ATCC 33331	SFLA_RS04490	SFLA_RS26395	SFLA_RS14085	SFLA_RS18365	SFLA_RS12045
Streptomyces fulvissimus DSM 40593	SFUL_1225	SFUL_1054	SFUL_1903	SFUL_2836	SFUL_4589
Streptomyces griseus NBRC 13350	SGR_1186	SGR_5993	SGR_3993	SGR_4295	SGR_2745
Streptomyces hygroscopicus jinggangensis	SHJG_RS17655	SHJG_RS15085	SHJG_RS11535	SHJG_RS23440	SHJG_RS29695
Streptomyces rapamycinicus NRRL 5491	M271_36375	M271_10530	M271_27400	M271_19940	M271_28285
Streptomyces scabiei 87.22	SCAB_RS32925	SCAB_RS35720	SCAB_RS23810	SCAB_53121	SCAB_RS17075
Streptomyces violaceusniger Tu 4113	Strvi_7083	Strvi_3001	Strvi_3450	Strvi_1100	Strvi_8677

[a] Strains are those have complete genomes.

[b] Make sure the chosen genes have no paralog in one genome before being used as RGs.

Table S4 List of primers used for real-time qRT-PCR in the present work

Primer name	Oligonucleotides (5' to 3')	Product length (bp)
SCO1453-F	GGCAGTACGACATCGGCAAGG	86
SCO1453-R	GCGCTCGAACACCCACAGG	
SCO2543-F	CTCCCGCTCATCGCCTACCA	168
SCO2543-R	GCCGTTGAAGAACAGGAAGTCGT	
SCO1962-F	CGGAGTTCTCCACCGTGCAGC	115
SCO1962-R	GGGTGATCGGCAGGAAGTAGTCG	
SCO4758-F	ATCACCGACCGGATGCCCTT	95
SCO4758-R	GCCGAGCCCCGCTTCTTC	
SCO1596-F	AAGCGGCTGATGCTGGAGAC	91
SCO1596-R	CGGAGTCGATCCGGGAGAT	
SCO6185-F	TGCACGCCCACTTCTGGATGTC	147
SCO6185-R	CGATGCGCTCGTAGGGGCTG	
SCO1544-F	TCGAGGTCGCCCGGGAACT	140
SCO1544-R	GATCACGTAGGTGGGGGTGCC	
SCO0710-F	TGTCCGCCCTCCGCTCCGTGTCC	172
SCO0710-R	TCCAGGACCGTGTCGCCGTAG	
SCO0301-F	CGGCACCGAACGGCATCTC	65
SCO0301-R	CTGCGTCAACTCGCTGAACCACA	
SCO3183-F	GGGCACCCTCGCGCTCC	96
SCO3183-R	TACTCGCCCCAGTCCAGGTCG	
SCO2742-F	CCCACCGCAACCTGCTCTTCA	101
SCO2742-R	GACTCCCGCCCACTTCAGCC	
SCO6218-F	CGTTCGTCAGCCCCTTCC	108
SCO6218-R	CCTCGTAGCCGCCGTAGTC	
SCO1519-F	GTCCGCGACTACGCCCAGGTCAAGG	203
SCO1519-R	ACTGTCTCACGCTCCTCCCCCACGG	
hrdB-F	CCTCCGCCTGGTGGTCTCG	132
hrdB-R	AACTTGTAGCCCTTGGTGTAGTCGAAC	
SAV_6806-F	CGAGGAGCTGGACCGTAAGGGAGA	176
SAV_6806-R	CGATGACATAGGTGGGGGTGCC	
SAV_6164-F	GAGCGCATCGCCGAGGTGA	108
SAV_6164-R	GACGTACGCGTGGTCGGACAGT	
SAV_4988-F	CCCCGAGGAGAAGCGGAAGAT	182
SAV_4988-R	CGTTGTGGTGGGACTTGATGTTG	
SAV_3674-F	CCTTGTGGTGAGAAGCCGGTTG	218
SAV_3674-R	CGAGATCGTTGTCCGCCAGG	
SAV_4006-F	CCCTCATCCCGCTCTTCGC	182
SAV_4006-R	GGCGGCGAGTTTCTGAAGTCCT	
SAV_2444-F	CGGTCAAGGACTACCTCAAGCAGAT	168
SAV_2444-R	TCCTCGGCGATGATCTCCAGC	

SCLAV1201-F	CCGTGCGATACCCGCCTTGT	173
SCLAV1201-R	CGGGGCGGATCGTCTTCAAC	
SCLAV0772-F	CTGTCCTGCCCCGACTGCC	127
SCLAV0772-R	CCGACACATAGGAGTGCTTGTTCTTC	
SCLAV2320-F	TCGCCGCGTATGTCTCCGTCG	127
SCLAV2320-R	TACTCCCCCCAGTCCAGGTCGTTGT	
SCLAV3213-F	TCCACTCGCACTACTGGCTGTCG	109
SCLAV3213-R	GCGGCGTTCTTGACCTTGGC	
SCLAV3712-F	GACCCGGAGGAGAAGCGGAA	192
SCLAV3712-R	ATTGTGGTGGGACTTGATGTTGGC	
SCLAV4698-F	AGGCCCGCACCATCCGTATC	135
SCLAV4698-R	GGGTCATGTCGAGTTCCTTGGC	

References

1 Zhao, Y., Xiang, S., Dai, X. & Yang, K. A simplified diphenylamine colorimetric method for growth quantification. *Appl. Microbiol. Biotechnol.* **97**, 5069-5077, (2013).

2 Huang, J., Lih, C. J., Pan, K. H. & Cohen, S. N. Global analysis of growth phase responsive gene expression and regulation of antibiotic biosynthetic pathways in *Streptomyces coelicolor* using DNA microarrays. *Genes Dev.* **15**, 3183-3192, (2001).

3 Nieselt, K. *et al.* The dynamic architecture of the metabolic switch in *Streptomyces coelicolor*. *BMC Genomics* **11**, 10, (2010).

4 Waldvogel, E. *et al.* The PII protein GlnK is a pleiotropic regulator for morphological differentiation and secondary metabolism in *Streptomyces coelicolor*. *Appl. Microbiol. Biotechnol.* **92**, 1219-1236, (2011).

5 Thomas, L. *et al.* Metabolic switches and adaptations deduced from the proteomes of *Streptomyces coelicolor* wild type and *phoP* mutant grown in batch culture. *Mol. Cell Proteomics* **11**, (2012).
